# An Investigation Into What Factors Influence Patterns of Clinical Presentation in Adult-Onset Celiac Disease

**DOI:** 10.7759/cureus.21924

**Published:** 2022-02-05

**Authors:** Katie O'Shaughnessy, William Stack, Triona Hayes, Elizabeth Kenny, Lucina Jackson

**Affiliations:** 1 School of Medicine, University College Cork, Cork, IRL; 2 Department of Gastroenterology, Bon Secours Hospital, Cork, IRL; 3 Department of Pathology, Bon Secours Hospital, Cork, IRL; 4 Department of Gastroenterology, Cork University Hospital, Cork, IRL

**Keywords:** marsh score, gut-brain axis, depression, anxiety, celiac disease

## Abstract

Introduction

Anxiety and depression are common in patients with celiac disease (CD), and many psychosocial explanations have been considered. However, as the gut-brain axis is becoming increasingly understood, biological mechanisms have been proposed, including vitamin or mineral deficiencies and gut inflammation.

Aim

To investigate associations between anxiety/depression and symptom severity, vitamin status, and gut inflammation in untreated adult patients presenting with a serologic indication of celiac disease.

Methods

The Hospital Anxiety and Depression Scale (HADS), Celiac Symptom Index (CSI), and Perceived Stress Scale (PSS) questionnaires were administered to 17 patients over a 14-month period. Duodenal biopsies were obtained to determine histological Marsh scores. Iron, B12, folate, vitamin D, and thyroid function tests were reviewed.

Results

HADS-Anxiety (HADS-A) scores correlated with symptom severity (rs = 0.62, P = 0.008), but not with any hematological investigations or degree of intestinal inflammation. No patients scored highly for depression. Iron deficiency was the most common deficiency observed (n = 6). Greater symptomatology was associated with female sex (females versus males: average CSI scores, 32.1 versus 23.6; t17 = 2.1, P < 0.05), younger age at presentation (rs = -0.55, P = 0.02), and lower Marsh score (Marsh 0 versus Marsh 3C: mean scores, 36 versus 24.5; t5 = 6.2, P = 0.009).

Conclusions

The anxiety experienced by patients with CD at presentation is likely a reactive form due to gastrointestinal symptoms rather than a biological process specific to CD. Older patients tend to present less symptomatically, highlighting the need for screening of at-risk individuals. The degree of villous atrophy does not correlate well with clinical presentation. Highly symptomatic patients should be screened for anxiety at presentation.

## Introduction

Celiac disease (CD) is a chronic small intestinal immune-mediated enteropathy precipitated by exposure to dietary gluten in genetically predisposed individuals [1]. It has an estimated prevalence of approximately 1% in those of European ancestry and is more common in the Irish population [2]. Historically, the disease has been associated with infants; however, many individuals present later in life [3]. While a number of patients experience the “classical” gastrointestinal symptoms of abdominal pain and diarrhea, as well as weight loss and malabsorption, a significant number of patients present solely with extraintestinal symptoms or may be entirely asymptomatic [4]. The only treatment for CD is a lifelong gluten-free diet (GFD), which aims to decrease symptoms and achieve mucosal healing.

As with many other chronic conditions, CD has often been associated with comorbid anxiety or depressive disorders [5,6]. Several studies have been conducted assessing patients with established CD who are implementing a GFD. However, it has been shown that the restrictive nature of a GFD can have a highly negative psychosocial impact [5,7]. This represents a major confounding factor when evaluating psychiatric symptoms. Furthermore, no studies of this nature have been conducted in an Irish population. Many potential theories have been explored; however, there is a significant lack of research on the factors that may contribute to affective disorders in this patient group. Some researchers believe that comorbid anxiety and depression are solely due to a reduced quality of life experienced by patients with CD secondary to adverse physical symptoms, along with personal and social limitations due to dietary restrictions [8,9]. However, taking into account the growing evidence on the link between the gut and the brain, it is necessary to consider the biological mechanisms that may account for psychological distress.

The gut-brain axis refers to the bidirectional biochemical signaling that takes place between the gastrointestinal tract and the brain through neural, hormonal, and immunological mechanisms [10]. It has been suggested that this signaling pathway is disrupted in CD, accounting for anxiety and depression comorbidity. Many mechanisms can be considered, including malnutrition [11], mucosal inflammation [12], and alterations in the gut microbiome [5]. Vitamin deficiencies are known to be highly prevalent in newly diagnosed CD, and mucosal inflammation is a major diagnostic feature [13]. Pro-inflammatory cytokines are thought to be implicated in neuropsychiatric disorders [14], and it has been well established that interleukins 1 and 6 are elevated in the serum of patients with active celiac disease [15].

At present, it has been found that patients with CD often experience heightened levels of anxiety and/or depression prior to the commencement of a GFD. However, it is unclear if this is a reactive state to symptom severity, an organic process, or a combination of the two. This is further complicated by contrasting findings regarding the resolution of anxiety and depressive symptoms on a GFD [7,16]. Therefore, the aim of the study was to evaluate the prevalence of anxiety and depression in untreated patients with CD and investigate both the biological and psychological factors that may contribute. The factors to be assessed include vitamin status, gut mucosal inflammation, and symptom severity. In addition to investigating the potential biological causes of anxiety and depression, the study also aims to investigate factors related to the wide range of clinical presentations of CD, such as age, sex, and comorbid illness.

## Materials and methods

Patient selection

Study invitation letters and information leaflets were posted to all patients > 16 years old with a serological indication of celiac disease scheduled for endoscopy with biopsy in the Bon Secours Hospital (BSH), Cork. In an effort to increase sample size, patients meeting the same criteria in Cork University Hospital (CUH) attending the public celiac outpatient clinic were also invited to participate on arrival at the clinic. The letter included an invitation to participate and an information sheet detailing the aims of the study and ethical considerations. Patients < 16 years or those with an established diagnosis of celiac disease were not considered for inclusion in the study. There were no other exclusionary criteria. All patients gave their voluntary informed consent following a verbal and written explanation of the aims of the study.

Questionnaires

Following consent, patients attending BSH were encouraged to complete the study questionnaires while waiting for their endoscopy procedure. In some cases, where a patient did not fully complete the questionnaire, they brought it home and returned it to the hospital when complete. Patients participating on the CUH site were not scheduled for endoscopy on the same day and were asked to complete the questionnaires while waiting for their clinic appointment.

Medical Questionnaire

A self-developed medical questionnaire was used to gain information on medical and psychiatric comorbidities, medication use, and general health. The participating patients were also asked specifically about recent use of probiotics or antibiotics, as well as the deliberate exclusion of dietary gluten. The patients were also asked about comorbid dermatitis herpetiformis, smoking status, and recent stressful life events. The impetus for obtaining serologic testing for celiac disease was also determined.

Psychological Assessment

Psychological symptom evaluation was conducted using two validated questionnaires. Anxiety and depression screening was conducted using the Hospital Anxiety and Depression Scale (HADS). This was selected due to its simplicity and validity. The HADS questionnaire has been shown to be an effective method of screening for anxiety and depression in the nonpsychiatric outpatient setting. It was also viewed to be optimal for this study given the evidence that scores are unaffected by physical illness [17]. The questionnaire consists of seven items relating to anxiety and seven for depression, each rated on a four-point Likert scale from zero to three. This allows a score of up to 21 to be assigned to each section. A total of ≥11 in the items relating to anxiety (HADS-A) indicates a probable anxiety disorder, most likely generalized anxiety disorder. A score of ≥11 in the items relating to depression (HADS-D) indicates a likely depressive disorder. For individual anxiety and depression scores, a total of between 8 and 10 indicates a borderline case. Scores ≤ 7 in each section are considered normal.

Perceived stress was determined using the 10-item version of Cohens Perceived Stress Scale (PSS). This validated questionnaire is designed to assess the degree to which an individual regards situations in their life as being stressful over the previous month. It was chosen as it has proven to be a useful tool in assessing the relationship between psychological stress and physical disorders. It consists of 10 items rated on a five-point Likert scale from zero to four, with six of the 10 being negatively phrased. A total score ranging from 0 to 40 is calculated by reversing the score of the four positively phrased items and summing them with the six negatively phrased items. A total score of ≤13 indicates low perceived stress, while scores of ≥14 and ≥27 indicate moderate and high levels of perceived stress, respectively.

Symptom Severity

Symptom severity was determined using the Celiac Symptom Index (CSI) questionnaire. This was selected as it is the only available validated CD-specific symptom index [18]. The CSI assesses both disease-specific symptoms and general health, which make up 11 and five questions, respectively. Each of the 16 symptoms are scored from 1 to 5, with a score of 1 indicating that the specified symptom is never present and a score of 5 indicating that the symptom is present all of the time. Total scores range from 16 to 80. Overall scores < 30 are associated with a low symptom burden and high quality of life, while scores > 45 indicate highly active disease with a low quality of life.

Vitamin status

For participants attending BSH, routine blood tests were performed as standard care for all patients with suspected CD. Folate, iron, B12, and vitamin D levels were reviewed in order to evaluate for deficiencies due to malabsorption. T4 and TSH levels were also obtained to assess thyroid function due to the increased risk for further autoimmune conditions in this population and the potential confounding factor of thyrotoxicosis manifesting with symptoms of anxiety. In the case of patients attending CUH, these blood tests were carried out by their general practitioner prior to them attending the clinic. The results of these tests were obtained on the CUH database and inputted to Statistical Package for the Social Sciences (SPSS) version 25 (IBM Corporation, Armonk, NY, USA).

Mucosal inflammation

The endoscopy procedure was carried out by the consulting gastroenterologist in the routine manner, with biopsies taken from the gastric antrum and the duodenum as routine standard of care. Histology reports were obtained from the pathology department of the Bon Secours Hospital to confirm or disprove a diagnosis of celiac disease. A histopathologist was consulted in order to determine the Marsh score of each patient. The Marsh grading system categorizes histopathological lesions into four discrete categories: pre‐infiltrative (type 0), infiltrative (type 1), infiltrative‐hyperplastic (type 2), and flat‐destructive (type 3). Type 3 can be further subdivided into a, b, or c subtypes, indicating mild, marked, or total villous atrophy, respectively [13].

Statistical analyses

All statistical analyses were performed using the Statistical Package for the Social Sciences version 25. The results of all questionnaires and relevant blood test results were inputted to the SPSS, along with the histological Marsh score, determined by the consultant histopathologist. Patients with a histology report negative for CD were considered to be controls (non-CD group). Data from descriptive statistical analyses were presented as percentages for categorical variables and as means and standard deviations (SDs) for continuous data. Correlations were assessed using Spearman’s rho in the case of ordinal and continuous data. Pearson’s Chi-square test or the likelihood-ratio Chi-square statistic were used to examine the relationship between categorical variables. Differences between subgroups were assessed using independent samples t-tests. A P-value < 0.05 was considered statistically significant for all statistical tests performed.

## Results

A total of 22 eligible patients were identified over a 14-month period. Two patients declined the invitation, and a further three patients failed to return questionnaires. This resulted in a total of 17 participants with a positive serological screening for celiac disease. Three included patients attended CUH, and the remaining 14 attended BSH. Marsh scoring was completed by a consultant histopathologist in BSH, and the patients were classified into CD (n = 11) and non-CD (n = 3) groups. The status of the three CUH patients could not be determined as biopsies were not performed. These patients were subsequently excluded from the analysis regarding the comparison of patients with and without CD but were included in all other aspects due to the low sample size of the study.

A summary of the demographic and clinical characteristics of the study population is provided in Table 1. As expected with most autoimmune disorders, the majority of participants were female (n = 12, 70.6%). The mean age of participants was 43.2 years and was lower in non-CD (36 years) versus CD patients (45.6 years). Vitamin deficiency was the most commonly stated reason for a patients’ general practitioner performing celiac screening. All patients were euthyroid.

**Table 1:** Clinical and Demographic Characteristics of the Study Population

	All	CD	Non-CD
	(n = 17)	(n = 11)	(n = 3)
Sex			
Male	5	3	1
Female	12	8	2
Age			
Range	20–70	20–70	23–43
Mean (SD)	43.2 ± 17.6	45.6 ± 19.6	3 6 ± 11.3
Coexisting Chronic Illness			
Yes	8	5	1
No	9	6	2
Smoking Status			
Lifelong Nonsmoker	11	8	3
Current Smoker	3	1	0
Ex-smoker	3	2	0
Indication for Celiac Screening			
Unknown	5	5	0
GI symptoms	4	1	1
Vitamin Deficiency	3	3	0
Fatigue	2	0	1
Family History	2	1	1
Comorbid Autoimmune Disease	1	1	0
Recent Stressful Life Event			
Yes	3	2	1
No	14	9	2
Any Deficiency			
Yes	9	4	3
No	7	6	0
Iron	6	1	3
Folate	2	2	0
B12	1	0	0
Vitamin D	1	1	0

Psychological assessment

The mean HADS-A scores were higher in the CD (7.7 ± 5.1) versus the non-CD (6.3 ± 2.5) group. However, this difference did not reach statistical significance (P = 0.66). All three patients who scored highly for anxiety were patients with CD. Two of these three patients had a preexisting diagnosis of an anxiety disorder. Borderline scores for anxiety were found in two patients. No patients scored highly for depression.

Spearman’s correlation coefficient revealed an association between HADS-A scores and the level of perceived stress (rs = 0.73, P = 0.01), as well as depression scores (rs = 0.69, P = 0.002). The HADS-A scores were not significantly higher in those who reported a recent stressful life event or those with a coexisting chronic illness. Anxiety levels had a moderate positive correlation with symptom severity (rs = 0.62, P = 0.008). Partial correlation analyses were used to further explore this relationship and revealed that this association was still applicable when a recent stressful life event and preexisting diagnosis of anxiety were controlled for (R = 0.75, P = 0.001). A summary of questionnaire results can be viewed in Table 2.

**Table 2:** HADS-A, HADS-D, PSS, and CSI Questionnaire Scores HADS-A: Hospital Anxiety and Depression Scale–Anxiety score; HADS-D: Hospital Anxiety and Depression Scale–Depression score; PSS: Perceived Stress Scale score; CSI: Celiac Symptom Index score

	All	CD	Non-CD
Questionnaire	N = 17	N = 11	N = 3
HADS-A	6.7 ± 4.6	7.7 ± 5.1	6.3 ± 2.5
Low	12	7	2
Borderline	2	1	1
Anxious	3	3	0
HADS-D	3.1 ± 2.5	2.9 ± 2.2	4.3 ± 3.5
Low	16	11	2
Borderline	1	0	1
Depressed	0	0	0
PSS	14.2 ± 7.2	14 ± 7.3	9.5 ± 14.5
Low	7	5	1
Moderate	10	6	2
Severe	0	0	0
CSI	29.6 ± 8.8	27.9 ± 9.2	36 ± 2
Low	9	8	0
Moderate	7	2	3
Severe	1	1	0

Symptom severity

An independent samples t-test was used to compare the mean CSI scores by diagnosis and gender. The mean CSI scores were found to be higher on average in the non-CD group, but this did not reach statistical significance (non-CD versus CD: average scores, 36 versus 27.9; t14 = 1.5, P = 0.17). Female patients generally reported higher symptomatology than male patients (females versus males: average scores, 32.1 versus 23.6; t17 = 2.1, P < 0.05). Partial correlation was used to control for a preexisting diagnosis of anxiety and reported recent stressful life events. This revealed that the association between gender and symptom severity was still applicable with these covariates accounted for (R = 0.5, P = 0.05). Spearman’s correlation coefficient demonstrated a moderate negative correlation between age at presentation and symptom severity (rs = -0.55, P = 0.02), indicating that younger patients were more symptomatic than their older counterparts. A detailed breakdown of symptomatology can be viewed in Table 3.

**Table 3:** Celiac Symptom Index Scores *Computed test statistic for equality of means **Two-tailed significance corresponding to the given test statistic

Symptom	Mean Item Score (range: 1–5)			Independent Samples Test	
	All (n = 17)	CD (n = 11)	Non-CD (n = 3)	T-Test Value*	P-value**
Abdominal Pain	2.01	1.81	2.67	-1.406	0.185
Nausea	1.65	1.64	2	-5.07	0.621
Rumbling	2.12	2	1.67	0.602	0.558
Bloating	2.29	2	3	-1.421	0.181
Diarrhea	1.41	1.18	1.67	-1.699	0.115
Tenesmus	2.06	2.09	2.33	-0.35	0.732
Hunger Pains	1.59	1.81	1.33	0.735	0.476
Fatigue	2.59	2.64	3	-0.475	0.643
Headache	1.47	1.45	1.67	-0.31	0.783
Cravings	1.71	1.81	2	-0.312	0.761
Anorexia	1.35	1.27	2	-0.156	0.146

Vitamin status

No significant differences were observed between the CD and non-CD groups in terms of any one vitamin deficiency. Nine patients suffered from any one deficiency. Iron deficiency was most commonly detected (n = 6), with all three non-CD patients being affected. Blood tests for all parameters were not available for every patient. No relationship was found between HADS-A, HADS-D, PSS, or CSI scores and serological levels of vitamin D, ferritin, folate, or B12.

Mucosal inflammation

**Figure 1:**
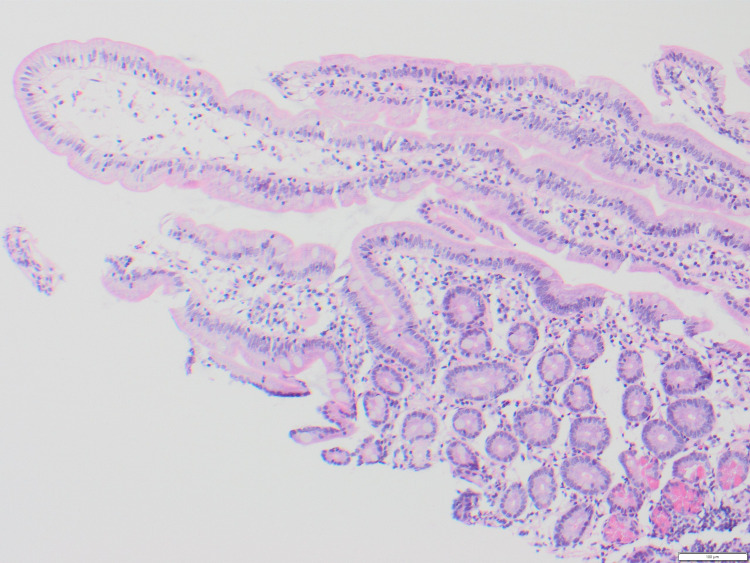
Marsh 0 on Duodenal Biopsy (H&E Stain)

**Figure 2:**
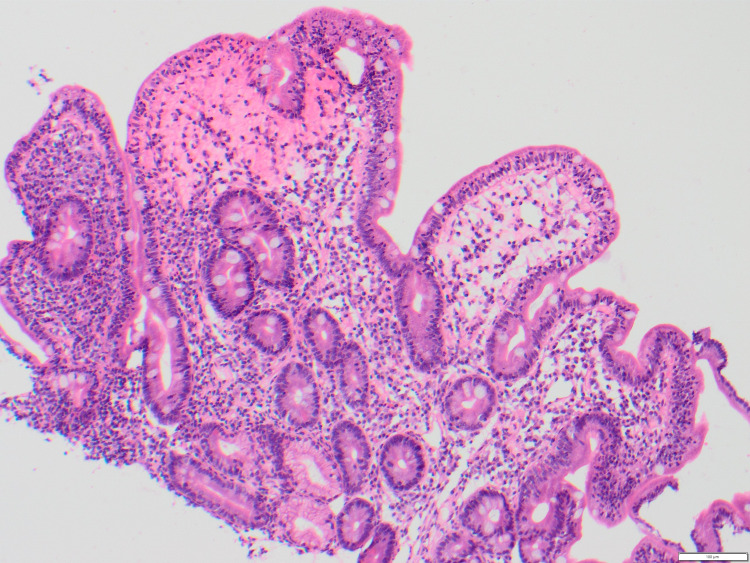
Marsh 3A on Duodenal Biopsy (H&E Stain)

**Figure 3:**
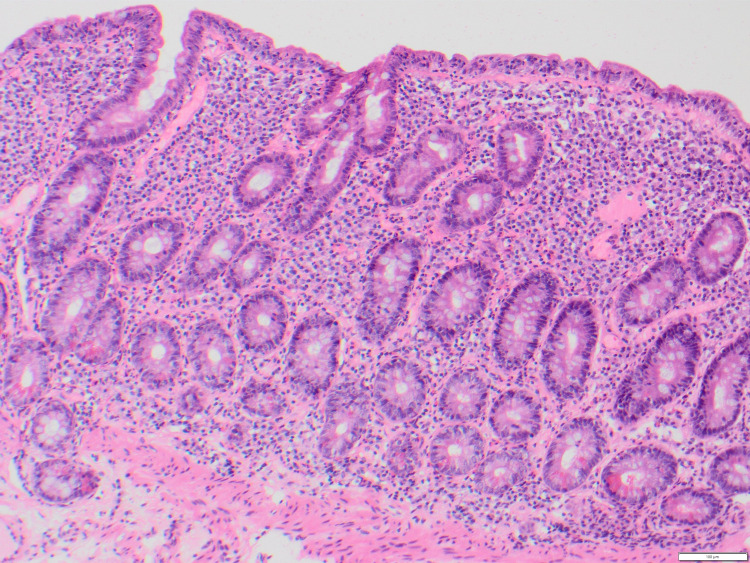
Marsh 3C on Duodenal Biopsy (H&E Stain)

Marsh scoring was completed for each patient with available biopsies (n = 14), as well as commentary on the level of active inflammation present. Three patients had a Marsh score of 0 (Figure 1), indicating a negative biopsy for CD. One patient had a Marsh score of 2, with this patient also reporting coexistent dermatitis herpetiformis. A Marsh score of 3A (Figure 2) was most commonly reported (n = 6), indicating mild villous atrophy. A Marsh score of 3B (n = 2) and 3C (n = 2) (Figure 3) were also reported, indicating marked and complete villous atrophy, respectively. The level of active inflammation present was classified into absent, mild, moderate, and severe subtypes in order to account for any other inflammatory process present that may be unrelated to CD. However, no patients who were negative for CD had active inflammation on histopathological examination. No relationship was found between Marsh scores and PSS, HADS-A, or HADS-D scores. An independent samples t-test was used to compare Marsh scores with mean CSI scores (Marsh 0 versus Marsh 3C: average scores, 36 versus 24.5; t5 = 6.2, P = 0.009), indicating that those with the highest Marsh score of 3C suffered less gastrointestinal symptoms than those without CD or any source of active gastrointestinal inflammation.

## Discussion

This is the first study assessing the biological factors relating to anxiety and depression in an Irish population of patients with CD. When compared with the limited number of studies assessing psychological symptoms at diagnosis, several findings are supported.

Despite not seeing a statistically significant difference in anxiety levels between CD and non-CD patients in our study, the mean anxiety scores were higher in the CD group than in the non-CD group (7.7 ± 5.1 versus 6.3 ± 2.5). This is in accordance with Addolorato et al., who found that patients with CD had higher mean anxiety scores at the time of diagnosis when compared with a non-CD group (IBD patients in this case) [19]. Another more recent study by Addolorato et al. examining state and trait anxiety in patients with CD also found high levels of state anxiety at diagnosis [20]. A further study investigating presentations in general practice found anxiety and/or depression to be a predictive factor for a subsequent CD diagnosis (OR = 2.5, 95% CI = 1.1-5.7, P = 0.031) [21]. In terms of factors associated with anxiety, we found a significant correlation with symptom severity (rs = 0.62, P = 0.008), which has been previously suggested to be a major contributing factor [7,19].

Although we did not observe a statistically significant difference in anxiety scores when comparing by gender (possibly due to the limited sample size of the study), the mean HADS-A scores were significantly higher in female versus male patients (7.6 ± 4.5 versus 4.6 ± 4.5). Due to the imbalance of male and female patients in our study (29% versus 71%, respectively), it is difficult to draw conclusions regarding this. Previous research has found anxiety levels to be higher in female patients with CD; however, these studies only included patients on a GFD [20,22].

In contrast to Addolorato et al., no patients in our cohort scored highly for depression at the time of diagnosis [7]. This may also support the observation of several larger studies that depression in patients with CD is a result of a restrictive lifestyle and, so, would only be seen after the commencement of a GFD [16,22].

Increased symptom severity was associated with female sex (females versus males: average CSI scores, 32.1 versus 23.6; t17 = 2.1, P < 0.05) and younger age at presentation (rs = -0.55, P = 0.02). Both findings are in agreement with the initial study validating the CSI as a disease-specific tool for CD [18]. Increased symptom severity in female patients at presentation could reflect a reciprocal relationship with anxiety or a potential overlap with functional gastrointestinal disorders [23]. Higher symptomatology in younger patients could indicate a more classical form of the disease in a younger age group [3], with older patients presenting silently or atypically [24].

Iron deficiency was the most frequent hematological abnormality detected, a common finding in adult-onset CD [25]. In agreement with a 2016 retrospective study investigating the association of psychological disorders with vitamin D deficiency, we found no correlations between serum vitamin D levels and anxiety scores [26]. Interestingly, this study also found that age at diagnosis was lower by approximately 10 years in those with a coexisting psychiatric disorder (P = 0.008). Our study did not observe any similar associations between age at diagnosis and anxiety or depression scores.

A negative association was found between the degree of villous atrophy and symptom severity. This is unsurprising, as it has been established that the degree of mucosal atrophy present on biopsy does not necessarily correlate with a clinical presentation [27]. As also found by Volta et al. in 2014, a high proportion of mild villous atrophy was observed, which may be the result of early detection due to screening in asymptomatic patients [28].

Limited research has been conducted examining the relationship between active mucosal inflammation, villous atrophy, and affective disorders in CD. While a 2011 retrospective study found an increased risk of suicide among patients with CD with active inflammation (HR = 1.96; 95% CI = 1.39-2.77), this may reflect poor GFD adherence secondary to psychological distress rather than depression as a direct result of an inflammatory process [29]. A 2018 study found that mucosal healing on follow-up biopsy was associated with a higher risk of future anxiety compared to persistent villous atrophy (HR = 1.49; 95% CI = 1.12-1.96) [16]. While this suggests that anxiety is a consequence of good GFD compliance, other research has proposed that affective disorders are a risk factor for poor adherence [30]. Given this information, further longitudinal studies assessing patients at presentation with follow-up of the cohort one-year post-GFD commencement would provide valuable information on the resolution or exacerbation of psychological symptoms. This would give further insight into whether good GFD adherence predicts continued psychological distress on follow-up or whether increased anxiety at presentation predicts poor GFD adherence.

## Conclusions

While this study cannot rule out contributing biological factors, our findings support the idea that anxiety in patients with CD at presentation is largely related to gastrointestinal symptom severity rather than features specific to the disease process. Patients who are highly symptomatic appear to be at risk for associated anxiety at the time of presentation. In terms of practical implications, this highlights a need for screening of at-risk patients at diagnosis, allowing for early implementation of psychological support that may improve GFD compliance. Furthermore, our findings support the need for low-threshold screening in older individuals due to a higher prevalence of subclinical diseases.

## Figures and Tables

**Figure 4:**
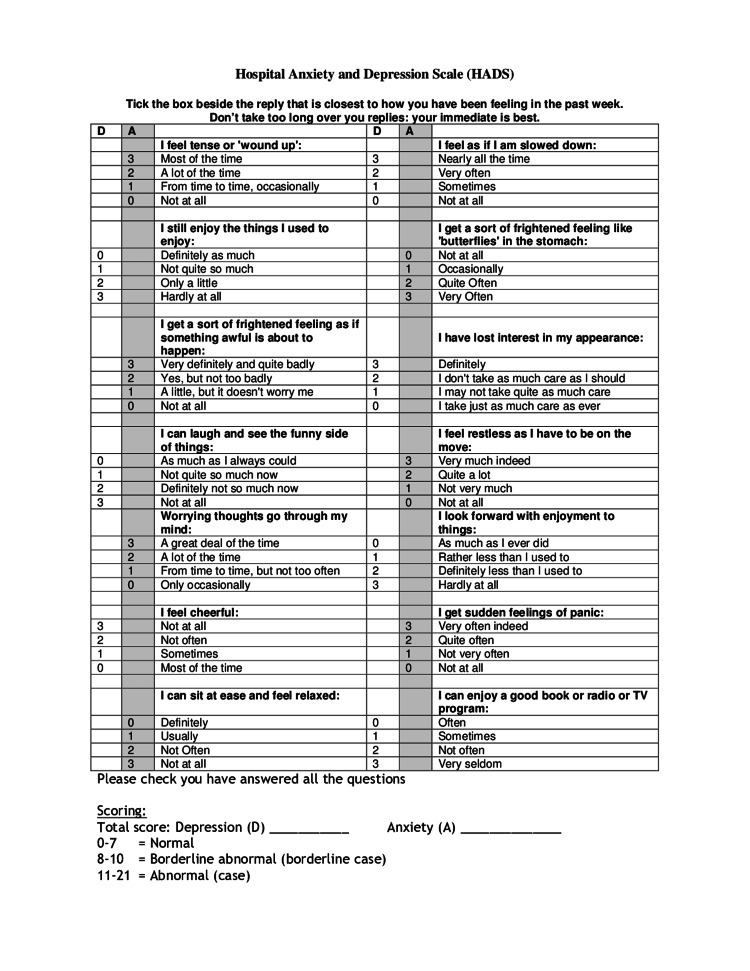
Hospital Anxiety and Depression Scale

**Figure 5:**
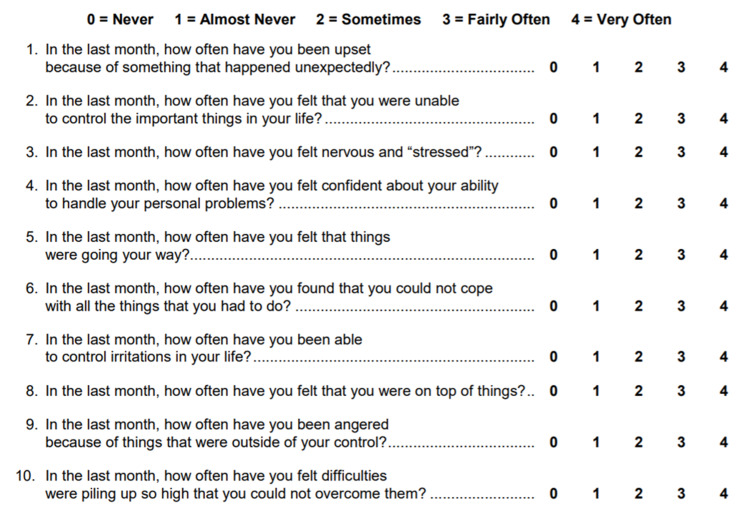
Perceived Stress Scale

**Figure 6:**
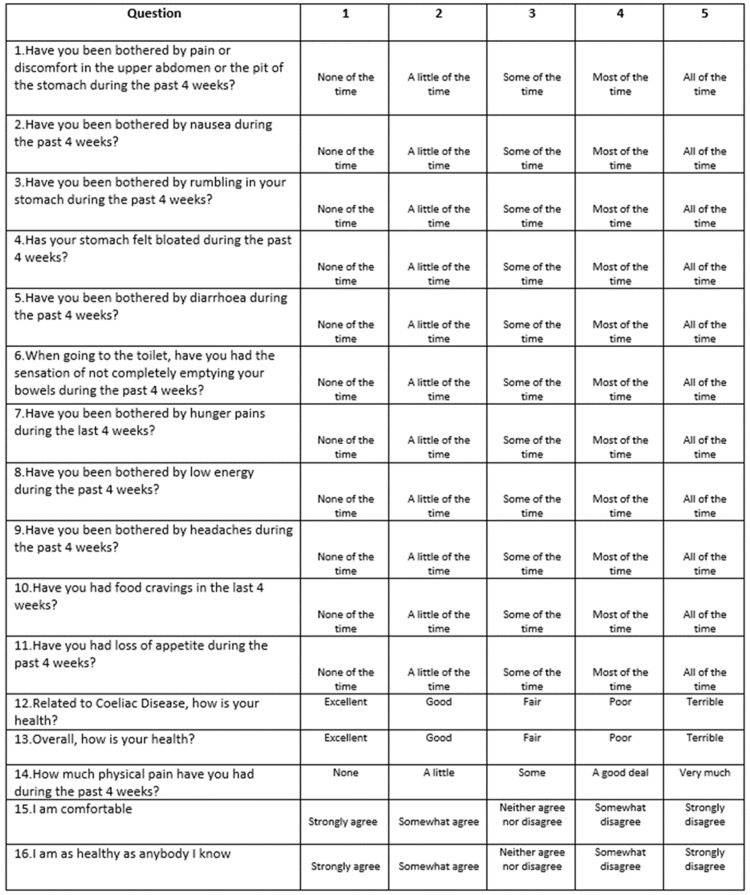
Celiac Symptom Index

## Appendices

The Hospital Anxiety and Depression Scale, Perceived Stress Scale, and Celiac Symptom Index questionnaires that were used in the present study are shown in Figure 4, Figure 5, and Figure 6, respectively.
